# Selection on Phalanx Development in the Evolution of the Bird Wing

**DOI:** 10.1093/molbev/msab150

**Published:** 2021-06-23

**Authors:** Merijn A G de Bakker, Wessel van der Vos, Kaylah de Jager, Wing Yu Chung, Donald A Fowler, Esther Dondorp, Stephan N F Spiekman, Keng Yih Chew, Bing Xie, Rafael Jiménez, Constanze Bickelmann, Shigeru Kuratani, Radim Blazek, Peter Kondrashov, Marilyn B Renfree, Michael K Richardson

**Affiliations:** 1 Animal Science & Health, Institute of Biology Leiden (IBL), Leiden University, Leiden, The Netherlands; 2 Museum für Naturkunde, Leibniz-Institut für Evolutions- und Biodiversitätsforschung, Berlin, Germany; 3 Naturalis Biodiversity Center, Leiden, The Netherlands; 4 Paläontologisches Institut und Museum, Universität Zürich, Zürich, Switzerland; 5 Departamento de Genética, Universidad de Granada, Lab 127 Centro de Investigación Biomédica, Granada, Spain; 6 Laboratory for Evolutionary Morphology, RIKEN Center for Biosystems Dynamics Research, Kobe, Japan; 7 RIKEN Cluster for Pioneering Research, Kobe, Japan; 8 Institute of Vertebrate Biology, Czech Academy of Sciences, Brno, Czech Republic; 9 Kirksville College of Osteopathic Medicine, A. T. Still University of Health Sciences, Kirksville, MO, USA; 10 School of BioSciences, The University of Melbourne, Melbourne, VIC, Australia

**Keywords:** limb development, evo-devo, hox genes, apoptosis, phalanx-forming region, frameshift theory, bird, reptile

## Abstract

The frameshift hypothesis is a widely accepted model of bird wing evolution. This hypothesis postulates a shift in positional values, or molecular-developmental identity, that caused a change in digit phenotype. The hypothesis synthesized developmental and paleontological data on wing digit homology. The “most anterior digit” (MAD) hypothesis presents an alternative view based on changes in transcriptional regulation in the limb. The molecular evidence for both hypotheses is that the MAD expresses *Hoxd13* but not *Hoxd11* and *Hoxd12*. This digit I “signature” is thought to characterize all amniotes. Here, we studied *Hoxd* expression patterns in a phylogenetic sample of 18 amniotes. Instead of a conserved molecular signature in digit I, we find wide variation of *Hoxd11*, *Hoxd12*, and *Hoxd13* expression in digit I. Patterns of apoptosis, and *Sox9* expression, a marker of the phalanx-forming region, suggest that phalanges were lost from wing digit IV because of early arrest of the phalanx-forming region followed by cell death. Finally, we show that multiple amniote lineages lost phalanges with no frameshift. Our findings suggest that the bird wing evolved by targeted loss of phalanges under selection. Consistent with our view, some recent phylogenies based on dinosaur fossils eliminate the need to postulate a frameshift in the first place. We suggest that the phenotype of the *Archaeopteryx lithographica* wing is also consistent with phalanx loss. More broadly, our results support a gradualist model of evolution based on tinkering with developmental gene expression.

## Introduction

The influential “frameshift” hypothesis postulates an evolutionary change in the phenotype of one or more digits in the lineage leading to birds (Wagner and Gauthier 1999; Stewart et al. 2019). The hypothesis aimed to reconcile conflicting data from developmental biology (Welten et al. 2005; Richardson 2012; de Bakker et al. 2013) and paleontology (Wagner and Gauthier 1999) about the homologies of the wing digits. Developmental data suggest an evolutionary loss of digits I and V from the ancestral pentadactyl forelimb (KundráT et al. 2002). For example, putative precartilage domains for these digits are seen transiently in the chicken and ostrich embryo wing bud (KundráT et al. 2002; Welten et al. 2005; de Bakker et al. 2013).

Paleontological data, by contrast, point to a pattern of reduction and loss affecting digits IV and V in archosaurs, the clade which includes birds. The basal theropods *Herrerasaurus ischigualastensis* (Sereno 1993), *Heterodontosaurus tucki*, and some other Heterodontosauridae in which the manus is preserved (Santa Luca et al. 1976; Santa Luca 1980; Sereno 2012), show reduction of forelimb digits IV and V. Digit reduction is reflected in the relatively small size, and loss of, skeletal elements; it suggests that the adult digits of the avian wing are homologous with ancestral digits I–II–III (Wagner and Gauthier 1999; Barta et al. 2018; Towers 2018). However, a recent dinosaur phylogeny based on an extensive fossils data set placed *Herrerasaurus* and Heterodontosauridae outside the lineage leading to birds as did the re-analysis of the same data set (Baron et al. 2017; Langer et al. 2017).

Evidence that has been cited in support of a frameshift includes a molecular signature (lack of *Hoxd11* and *Hoxd12* expression in digit I) that is stable across amniotes (Vargas and Fallon 2005; Vargas et al. 2008; Woltering and Duboule 2010; Salinas-Saavedra et al. 2014). A recent transcriptomics-based study confirmed this digit I molecular signature (Stewart et al. 2019). It was found to consist of lower expression of *Hoxd11*, *Hoxd12, Sall1* compared with other digits. In the chicken hind- and mouse and alligator forelimb in that study, ten genes are differentially expressed in digit I. That study also proposed a modified version of the frameshift in which only the anterior digit is affected, yielding a I-III-IV pattern of digit homologies in the bird wing. The conclusion was based on data from four species: the mouse, alligator, green anole, and chicken (Stewart et al. 2019).

An alternative explanation for the distinctive expression profile of digit I is “the most anterior digit” (MAD) hypothesis (Woltering and Duboule 2010). This hypothesis posits a lack of expression of *Hoxd10*, *Hoxd11*, and *Hoxd12* genes in digit I in amniotes. However, it takes into account the transcriptional landscape of the wrist or ankle (mesopodium), which is a low-hox zone; it suggests that digit I may have evolved from low-hox tissue through a gain in *Hoxa13* expression and reinforced expression of *Hoxd13* (Woltering and Duboule 2010). The MAD hypothesis posits that the unique morphology of digit I is due to “a reduced dosage of HOXD proteins” collectively (Woltering and Duboule 2010, pp. 529–530).

Both the frameshift and MAD hypotheses make predictions about the evolution of developmental gene expression. Specifically, they assume that the expression of *Hoxd13* in the MAD, coupled with a lack of expression of *Hoxd11* and *Hoxd12* in that digit, is stable across the amniotes. We have tested this prediction by analyzing the expression patterns of posterior *Hoxd* genes—but in a much larger sample of amniote species than previously studied. By using a comparative approach, we are following the advice of Cuvier who advocated the study of “experiments ready prepared by Nature” (Cuvier 1840, p. 15). In that spirit, we are studying species genetically modified by evolution. We have also examined cell death in the phalanx-forming region and made a survey of the patterns of digit loss in adult amniotes. Our comparative data complement the results of functional studies, and test their predictions in an evolutionary perspective. In summary, we are using gene expression patterns as markers of homology. In view of the large number of species studied here, we focused in particular on *Hoxd11*, *12*, and *13* because these are the most frequently used markers in the literature on digit I homologies. Also the protein sequences of *Hoxd11*, *Hoxd12*, and *Hoxd13* were aligned to look for sequences differences that might explain differences in their spatial expression during limb development.

## Results

### *Hoxd* Expression in Digits

**Fig. 1.**
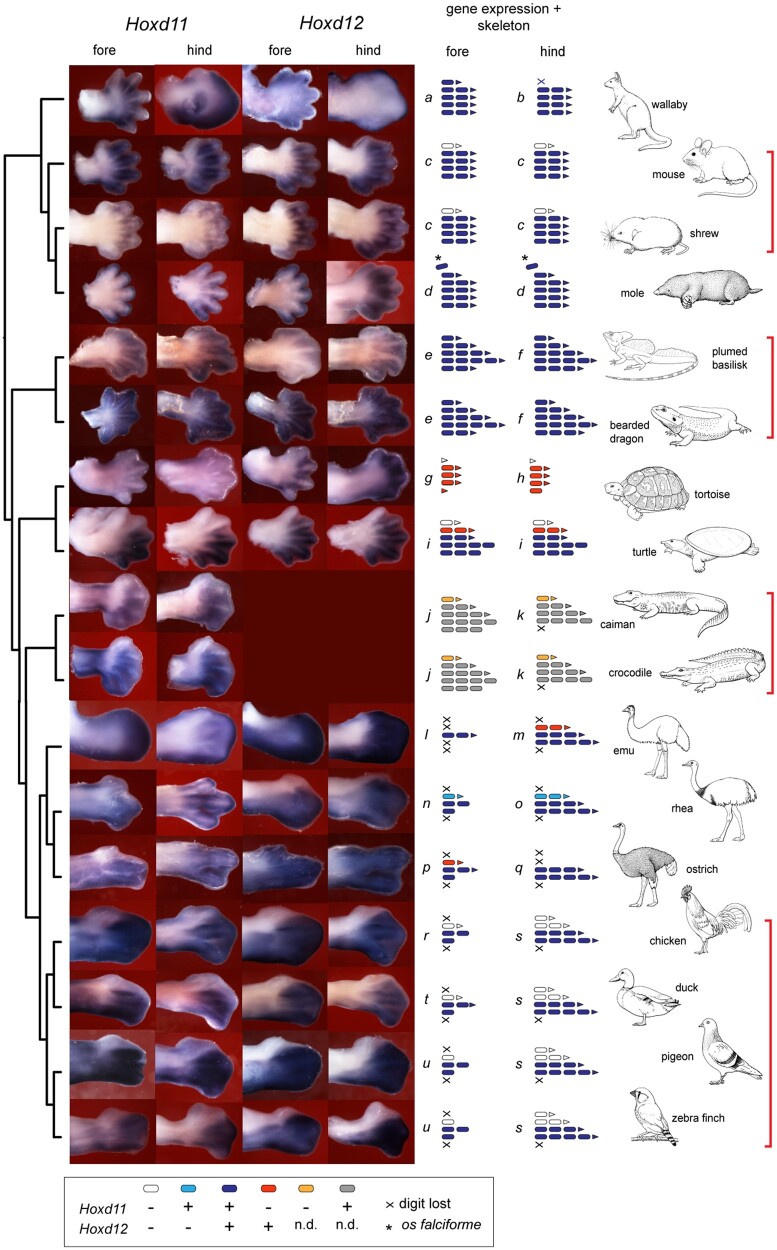
*Hoxd 11* and *Hoxd12* expression and skeletal phenotype in limb evolution. Photographs showing wholemount in situ hybridization of *Hoxd11* and *Hoxd12* genes at stages showing early crenation of the digital plate margin. Because of heterochrony between the forelimb and hindlimb, there are differences in stage; this is especially true for the emu. The right half of the figure shows schematic summaries of the molecular-morphological phenotype (i.e., the embryonic expression pattern of *Hoxd11* and *Hoxd12* and the associated adult digital and phalangeal formulae). Each unique phenotype is labeled *a–u*. The red brackets indicate clades sharing a conserved molecular-morphological phenotype (indicated in red brackets, right). The only exception to this conserved pattern was minor variations in the presence or absence of vestigial ungual phalanges in the Neognathae (lower red bracket). n.d., not determined. Schematized skeletal phenotypes are based on specimens in figure 8 and supplementary figure S4 and table S3, Supplementary Material online). The apparently diffuse expression of *Hoxd11* and *Hoxd12* in the mole and the shrew is not a failure of the probes; in fact, the probes are specific, as we show in the whole embryo in situs in supplementary figure S5, Supplementary Material online. Line drawings by Esmée Winkel.

The embryos examined are listed in supplementary table S1, Supplementary Material online. Figure 1 shows the patterns of expression of *Hoxd11* and *Hoxd12* in the forelimb and hindlimb in 17 amniote species, and includes a summary mapping of the expression patterns onto the limb skeletal patterns (fig. 1*a–u*). We could not amplify *Hoxd12* in the crocodilians, and there was no hybridization of our chicken *Hoxd12* probe to crocodilian embryos. In all species where it could be determined, the anterior boundary of *Hoxd11* and *Hoxd12* expression in the forelimb was the same as in the hindlimb.

In the forelimb and hindlimb of the shrew, mouse and the two crocodilians, there was no detectable expression of *Hoxd11* in digit I (fig. 1*c*, *j*, and *k*). Furthermore, there was no expression of *Hoxd12* in digit I of the mouse, shrew or tortoise (fig. 1*c*, *g*, and *h*). The MAD in the wing of the neognaths examined (chicken, duck, pigeon, and zebra finch) showed no expression of either *Hoxd12* or *Hoxd11* (fig. 1*r*–*u*). Although these expression patterns are consistent with the frameshift hypothesis, the remaining data are not.

In the developing hindlimbs of the neognaths examined, *Hoxd11* and *Hoxd12* expression is lacking in digit I and in digit II (fig. 1*s*). This is consistent with fig. 5 in Huang et al. (2016) and fig. 6b in Stewart et al. (2019). The paleognaths examined (emu, rhea, and ostrich) show a different pattern. All three digits in the rhea forelimb and hindlimb express *Hoxd11*, whereas only digits III and IV show expression of *Hoxd12* (fig. 1*n* and *o*). In the ostrich wing, the MAD lacks *Hoxd11* expression (fig. 1*p*). In the emu hindlimb, digit II lacks *Hoxd11* expression (fig. 1*m*). In the forelimb and hindlimb of the emu and ostrich, all digits express *Hoxd12* (fig. 1*l*, *m, p*, and *q*). Note that the adult emu wing has only a single digit, the ostrich foot has two digits and the emu and rhea hindlimbs have three digits—all of which have unambiguous homologies according to published studies (Maxwell and Larsson 2007; Kundrát 2009; Schaller et al. 2011; de Bakker et al. 2013; de Almeida et al. 2015).

In the developing limbs of the Chinese soft-shelled turtle, there was no expression of *Hoxd11* in digits I and II, and no expression of *Hoxd12* in digit I (fig. 1*i*). In the strongly reduced digits of the tortoise only *Hoxd12* is expressed in the four posterior digits (fig. 1*g*). *Hoxd11* was not detected in the limb but in the same sample it was in the genital swellings. The bearded dragon and plumed basilisk show *Hoxd11* and *Hoxd12* expression in all five digits (fig. 1*e* and *f*). In the wallaby, both *Hoxd11* and *Hoxd12* are expressed in all five digits of the hand, and in all four digits of the foot (the foot lacks digit I; Chew et al. 2012; fig. 1*a* and *b*). The mole shows *Hoxd11* and *Hoxd12* expression in all five digits and in the extra digit-like element, the *os falciforme* (fig. 1*d*).

The variation that we find in the anterior expression boundaries of *Hoxd11* and *Hoxd12* is unlikely to be a random experimental artefact, because it is consistent within clades (as indicated by red brackets on the right in fig. 1). Within these four clades, both the skeletal phenotype and the expression boundaries of *Hoxd11* and *Hoxd12* are conserved. Furthermore, our patterns are consistent with the results of (Vargas et al. 2008; Stewart et al. 2019), which our study, show lack of expression of those two genes in digit I of the alligator, mouse and chick, and uniform expression across all digits in *Anolis*.

**Fig. 2.**
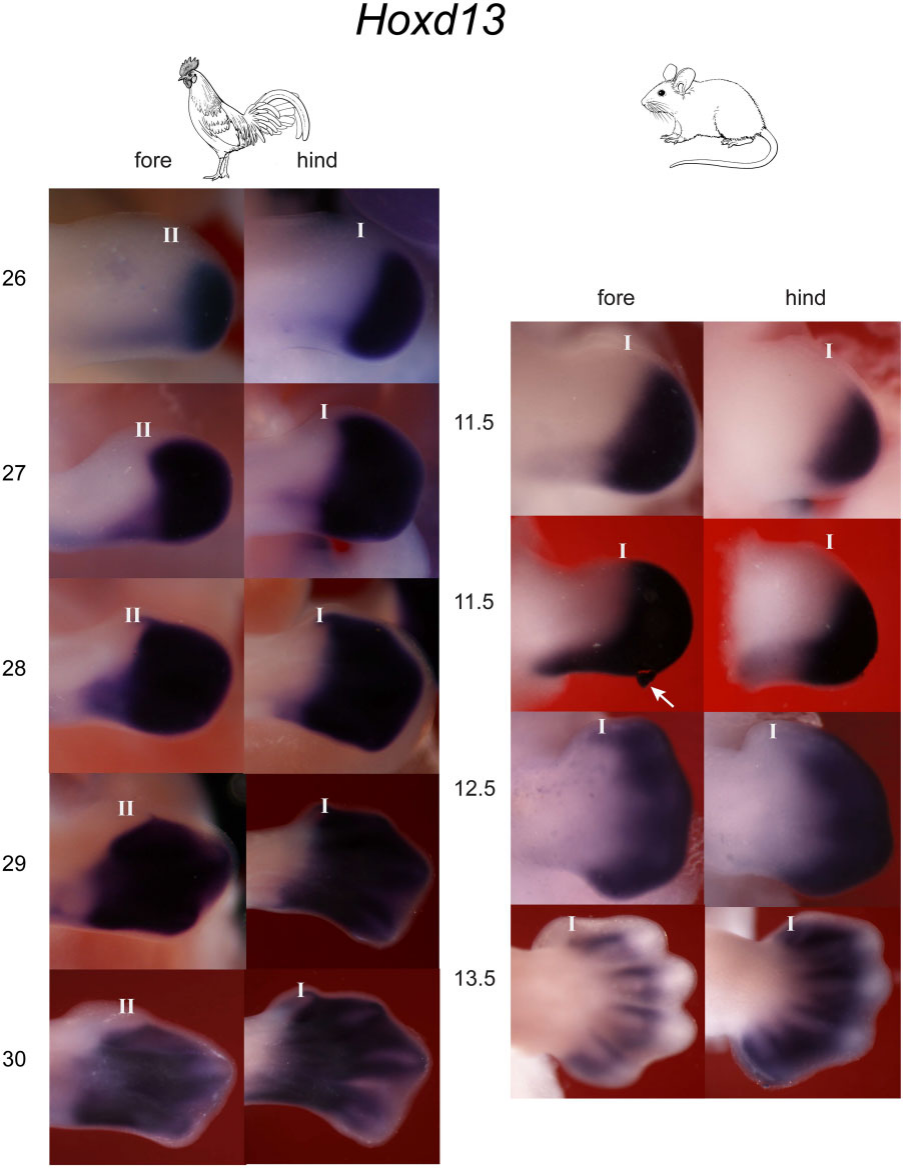
Stage-dependent *Hoxd13* expression in chicken and mouse embryo limbs. In the chicken (two columns, left), expression expands into all digits in the hindlimb, but not into digit II in the wing. Numbers on left indicate stages (Hamburger and Hamilton 1951). In the mouse (two columns, right). In the mouse a small domain of *Hoxd13* expression was seen in forelimb digit I at E12.5, but had disappeared by E13.5. Numbers on left indicate days of gestation; fore, forelimb; hind, hindlimb; arrow, artefact (damage); I, digit I; II, digit II. Please note that the stages in the chicken and mouse are not directly comparable because of heterochrony and other issues. Line drawing by Esmée Winkel.

**Fig. 3.**
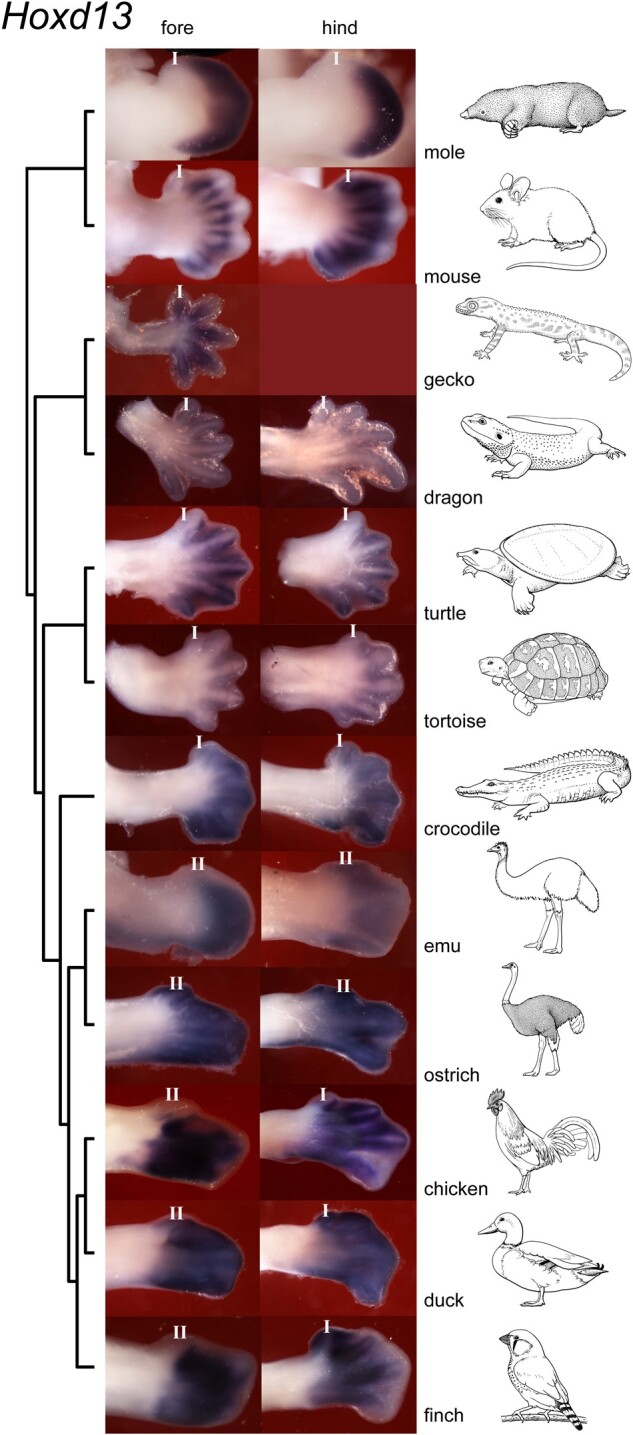
Hoxd13 expression in the developing limbs of 12 amniote species. Note that the expression domain extends across all digits, except in the case of the mole hindlimb, the mouse forelimb, the turtle forelimb and hindlimb, the emu forelimb, chicken forelimb and hindlimb, and the zebra finch hindlimb. We do not know whether this is simply because we did not capture the full anterior extent of the expression domain in the stages we studied. Line drawings by Esmée Winkel.

We also tested the hypothesis that *Hoxd13* is expressed in all five digits in amniotes (in second-phase *Hox* gene expression, when the domains expand to the autopod) (Chen et al. 2005; Woltering and Duboule 2010; Chew et al. 2012). We examined *Hoxd13* expression patterns in staged series of chick and mouse limbs. These series confirmed that *Hoxd13* is not strongly expressed in all five digits in both limbs in these species (fig. 2); rather, it shows only weak expression in mouse forelimb digit I, and lack of expression in chicken wing digit II (the MAD). In addition, we found that *Hoxd13* was not expressed in digit I in the mole forelimb and hindlimb, the turtle forelimb and hindlimb or in zebrafinch wing digit II, for these species, see figure 3. In summary, digit I does not have a stable molecular signature, as defined by *Hoxd11, Hoxd12*, and *Hoxd13* expression across amniotes.

In the protein alignment all three hoxd genes showed extremely high conservation of their homeodomain. The *Hoxd11* and *Hoxd12* protein sequences in amniotes are strongly conserved in both the amino and carboxyl terminus but the sequence in between these termini is variable, including insertions and deletions in some groups (supplementary figs. S1 and S2, Supplementary Material online). The three crocodilians studied (*Alligator mississippiensis, A. sinensis*, and *Crocodylus porosus*) have a 150+ amino acid deletion in their *Hoxd12* sequence, about half the normal *Hoxd12* protein length (supplementary fig. S2, Supplementary Material online). The *Hoxd13* alignment shows at first inspection a truncation of the N-terminus terminal but this is mainly due to incomplete sequences (supplementary fig. S3, Supplementary Material online).

### Premature Termination of Development in Reduced Digits

We now consider possible alternative mechanisms of phalanx loss and digit reduction. We begin with the functional morphology of the wing, because it is likely that this has been under selection in the lineage leading to birds.

The digits and metacarpus of the bird wing are functionally important because they carry flight feathers or primary remiges (we use the term “digit” here for the phalangeal part of the autopod). However, the three wing digits each carry different numbers of flight feathers (Humphrey and Clark 1961; Lucas and Stettenheim 1972) and have correspondingly different roles in flight. Digit II (the alula) carries four small flight feathers (Humphrey and Clark 1961; Lucas and Stettenheim 1972) and has crucial roles in certain flight modes (Carruthers et al. 2007), where it may act as a vortex generator (Lee et al. 2015). Digit III is the major functional digit and carries three or four large flight feathers on its phalanges (Humphrey and Clark 1961; Lucas and Stettenheim 1972). Digit IV, by contrast, has only a single phalanx with no claw (Richardson 2012), and bears only one flight feather (Humphrey and Clark 1961; Lucas and Stettenheim 1972). In summary, digit IV has become functionally and morphologically reduced.

In order to examine how digit IV became so reduced, we have studied the phalanx-forming region (Suzuki et al. 2008; Huang et al. 2016; Hiscock et al. 2017), a collection of *Sox9*-expressing mesenchyme cells at the tips of the digits, that is essential for digit growth and patterning (Suzuki et al. 2008; Huang et al. 2016; Hiscock et al. 2017). Because *Sox9* is expressed in the phalanx-forming region, we use it here as a marker. To look for programmed cell death (apoptosis), we developed a protocol for the whole mount staining of embryos using TUNEL (terminal deoxynucleotidyl transferase dUTP nick-end) (Gavrieli et al. 1992; Crowley et al. 2016), for details, see Materials and Methods.

**Fig. 4.**
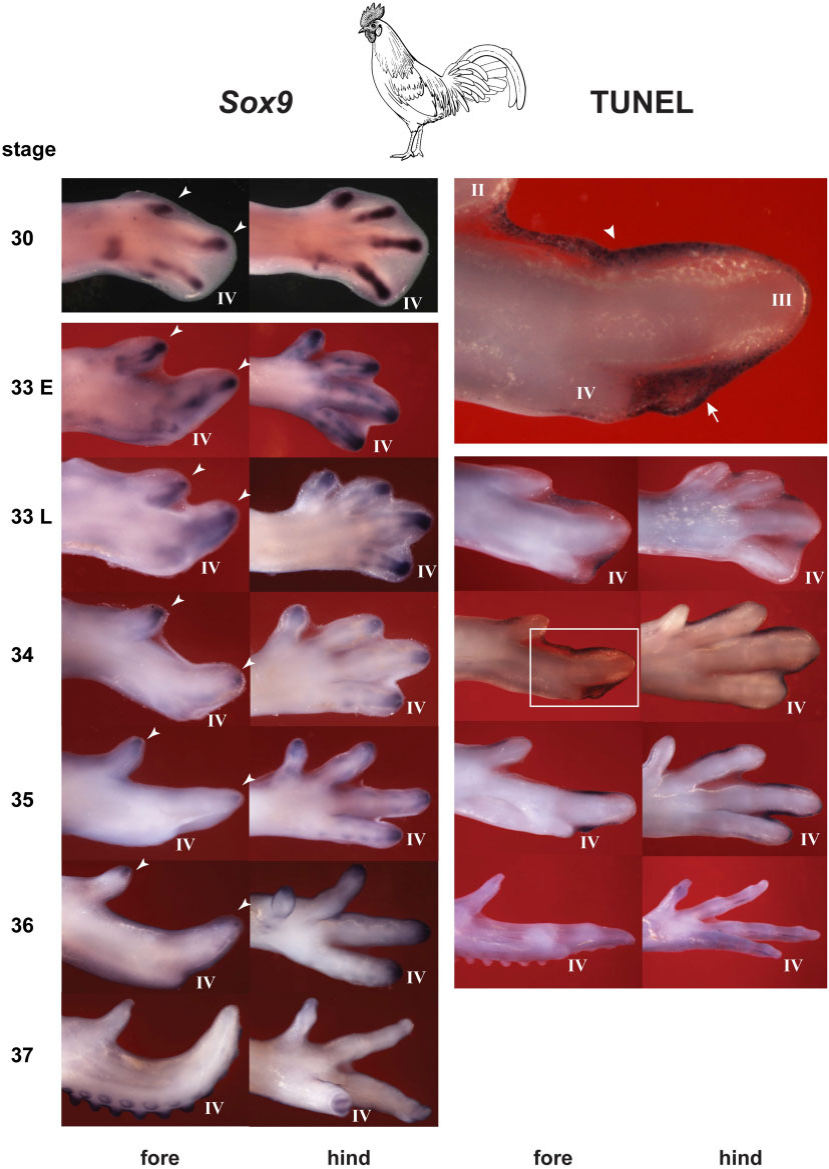
Chicken *Sox9* expression and TUNEL staining. Note that the phalanx-forming region is well-developed in wing digits II and III (arrowheads) but not in the late digit IV. The large figure at the top of the right column shows an enlargement of the boxed area in the stage 34 forelimb. TUNEL staining is visible in the interdigital mesenchyme (arrowhead) and in the mesenchyme at the tip of digit IV (arrow in enlargement; see also fig. 7). Notice that the strong expression in the phalanx forming region of wing digit IV at stage 30 has disappeared by stage 33 onward. The faint *Sox9* expression in the region of digit IV at stage 33 represents prechondrocytes, not the phalanx-forming region. The early termination of the phalanx-forming region in wing digit IV is in contrast to its persistence in the other two wing digits, and the four digits of the hindlimb. The same phenomenon is seen in the duck (fig. 5) and in the crocodile hindlimb digit V, which is vestigial in the adult skeleton. Line drawing by Esmée Winkel.

**Fig. 5.**
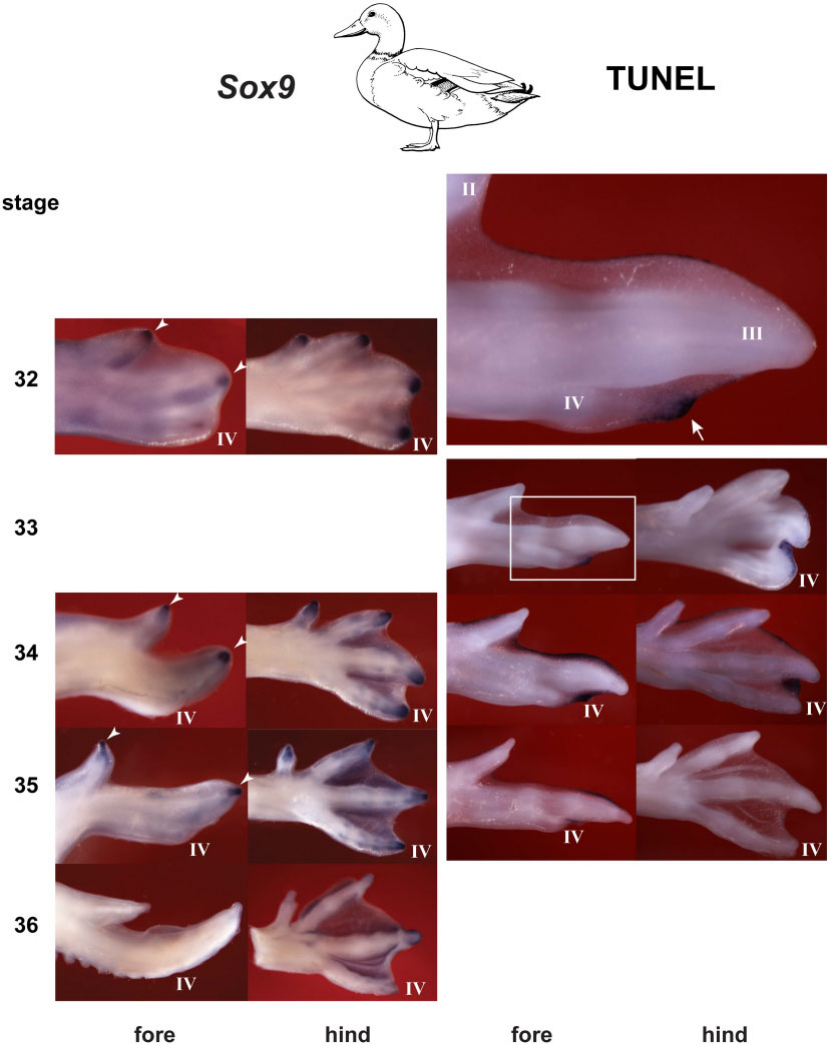
Duck *Sox9* expression and TUNEL staining. The large figure at the top of the right column shows an enlargement of the boxed area in the stage 33 wing. Note that the phalanx-forming region is well-developed in all wing digits II and III (arrowheads). The *Sox9* expression at stage 34 in digit IV is probably produced by differentiating prechondrocytes developing in the digit IV phalanx. TUNEL staining is present at the tip of digit IV in the wing (arrow). TUNEL staining in the interdigital mesenchyme is more restricted than in the chicken, especially in the hindlimb, which is webbed in the adult (compare fig. 4; see also fig. 7). Line drawing by Esmée Winkel.

**Fig. 6.**
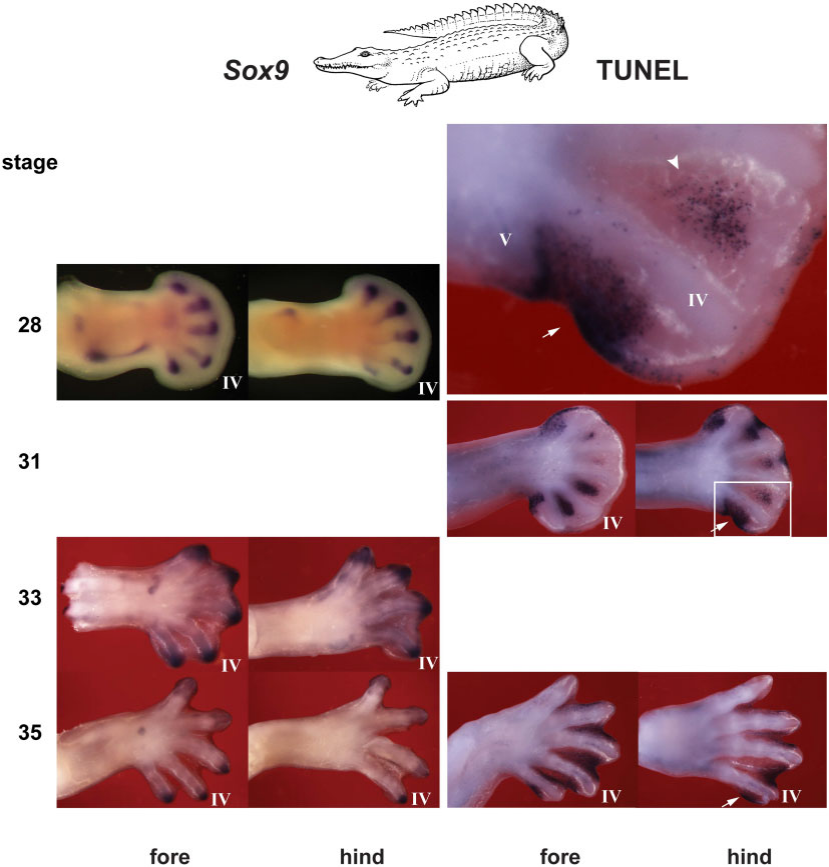
Nile crocodile *Sox9* expression and TUNEL staining. In this species, hindlimb digit V is vestigial (de Bakker et al. 2013). The large figure at the top of the right column shows an enlargement of the boxed area in the stage 31 hindlimb. *Sox9* expression shows that the phalanx-forming region is well-developed in hindlimb digits I–IV but not in digit V. The *Sox9* expression at stage 28 in digit V is probably produced by differentiating prechondrocytes of the vestigial digit V. In addition, there is a strong TUNEL staining in the prospective hindlimb digit V region (arrow), and also in regions of interdigital mesenchyme. Line drawing by Esmée Winkel.

We compared the chicken and duck, because of their strongly reduced forelimb digit IV, and the Nile crocodile because it also has a strongly reduced digit, namely hindlimb digit V. Wing digit IV has a metacarpal and a reduced phalanx with no claw (Richardson 2012; de Bakker et al. 2013). The crocodylian toe V is reduced to a single, vestigial bone, presumably the metatarsal bone with no claw (Müller and Alberch 1990; de Bakker et al. 2013). In the chicken (fig. 4) and duck (fig. 5), there is a well-developed phalanx-forming region at the tip of all wing digits, as indicated by strong *Sox9* hybridization. However, the phalanx forming region of wing digit IV disappears early compared with the other wing digits (figs. 4 and 5). After this, the only *Sox9* hybridization remaining in digit IV is in weak hybridization in the precartilage of the developing phalanx (for which *Sox9* is also a marker [Welten et al. 2005; Lorda-Diez et al. 2011; de Bakker et al. 2013]). The early disappearance of the phalanx forming region in digit IV in both birds is followed by strong TUNEL staining (figs. 4 and 5) indicating programmed cell death. In the crocodilian hindlimb digit V, we see a similar scenario: early arrest of the phalanx forming region followed by apoptosis (fig. 6).

These findings are consistent with a model in which individual digits are subject to selection for morphological reduction during evolution. They are also consistent with a mechanism of digit reduction based on targeted loss of the phalanx-forming region followed by localized apoptosis. As we show, these two mechanisms operate together in two different digits, in different clades and presumably under different selective regimes. This targeted reduction of single digits is not consistent with the predictions of the frameshift model (Wagner and Gauthier 1999), or its modified version (Stewart et al. 2019), since those models assume a change from one complete digit phenotype to another; nor do they account for changes in morphology due to functional reduction.

**Fig. 7.**
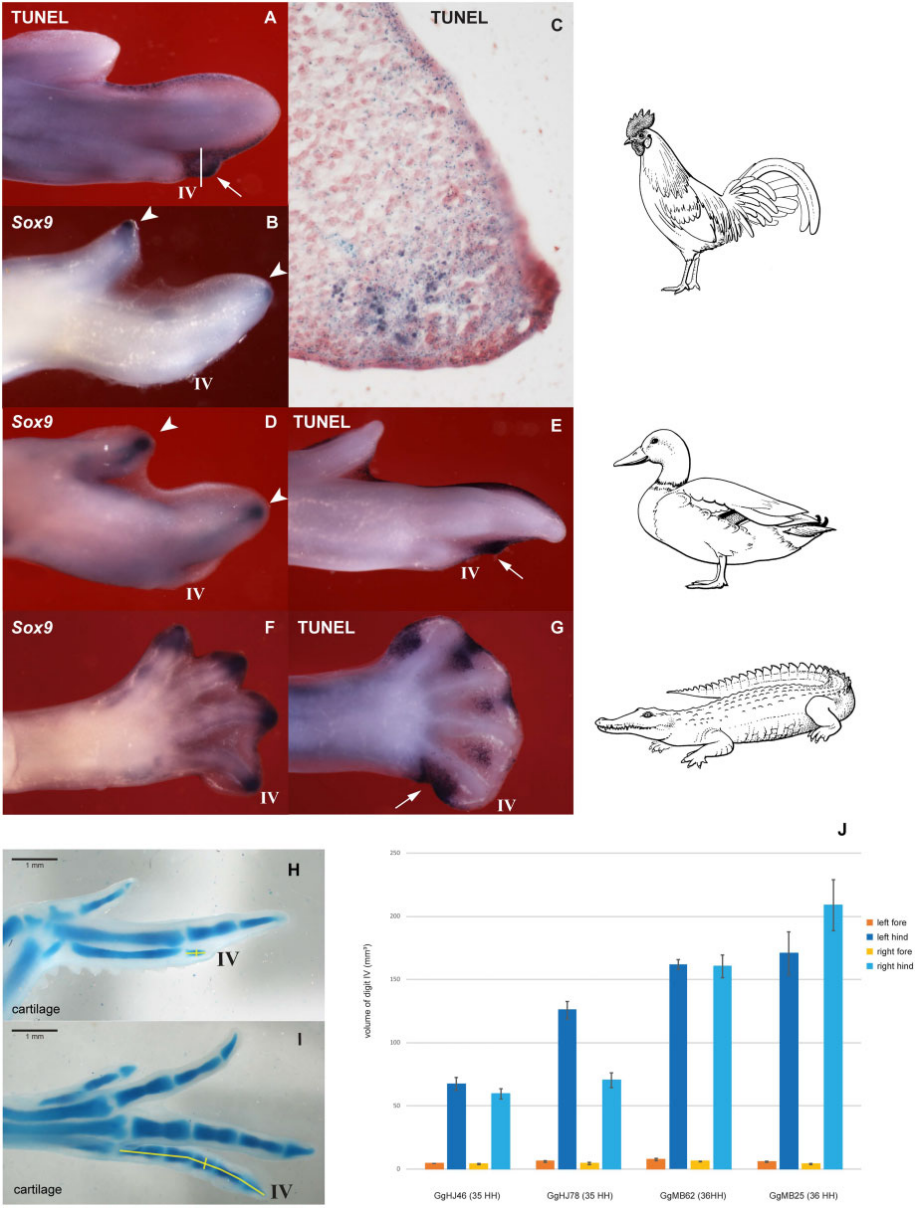
Summary of the phalanx-forming region, cell death, and tissue volume in digit IV. (*A*, *E*, *G*) TUNEL staining in the chicken, duck, and crocodile embryos; (*C*) Histological section showing TUNEL staining in a chicken from the digit IV in (white line in *A* shows the plane of section). (*B*, *D*, *F*) expression of *Sox9*, in the same species. *Sox9* is a marker of the phalanx-forming region (Suzuki et al. 2008; Huang et al. 2016; Hiscock et al. 2017). In both birds there is a well-developed phalanx-forming region (arrowheads) in wing digits II and III but not in digit IV. There is also strong TUNEL staining at the tip of digit IV. In the crocodile, the phalanx-forming region is well-developed in hindlimb digits I–IV; there is strong TUNEL staining (arrow) in the region of the vestigial digit V. (*H*, *I*) Cartilage wholemounts (Alcian blue stain) of chicken forelimb (*H*), and hindlimb (*I*), stage 36. Yellow lines demarcate the areas of digit IV measured for the analysis in (*J*). (*J*) Measurements of tissue volume in chick digit IV, forelimb and hindlimb, stages 35 and 36. For table of measurements see (supplementary table S4, Supplementary Material online). Line drawings by Esmée Winkel.

Loss of the *Sox9*-expressing phalanx-forming region, followed by cell death, has had dramatic effects on the phenotype of wing digit IV (compared with its counterpart in the hindlimb). Thus, in addition to phalanx loss, this digit has suffered a substantial size reduction (fig. 7*C*–*E*). Wing digit IV is 15× (stage 35) to 25× (stage 36) smaller in terms of tissue volume and has one phalanx instead of five (fig. 7*E*).

Together, the data in this section have consequences for the interpretation of fate-mapping studies (Tamura et al. 2011; Towers et al. 2011). Those studies reported differences, between the chick forelimb and hindlimb, in the origin of mesenchyme for digit IV. The results were interpreted as supporting a frameshift in the wing, relative to the hindlimb. However, a relatively simple, alternative explanation of these results is that wing digit IV represents a far smaller tissue domain than toe IV. Furthermore, the apoptosis that we have demonstrated in wing digit IV, and loss of phalanx-forming region, would explain why the polarizing region of the wing gives rise only to marginal soft tissue (Towers et al. 2011).

### Widespread Phalangeal Loss in Amniotes

**Fig. 8.**
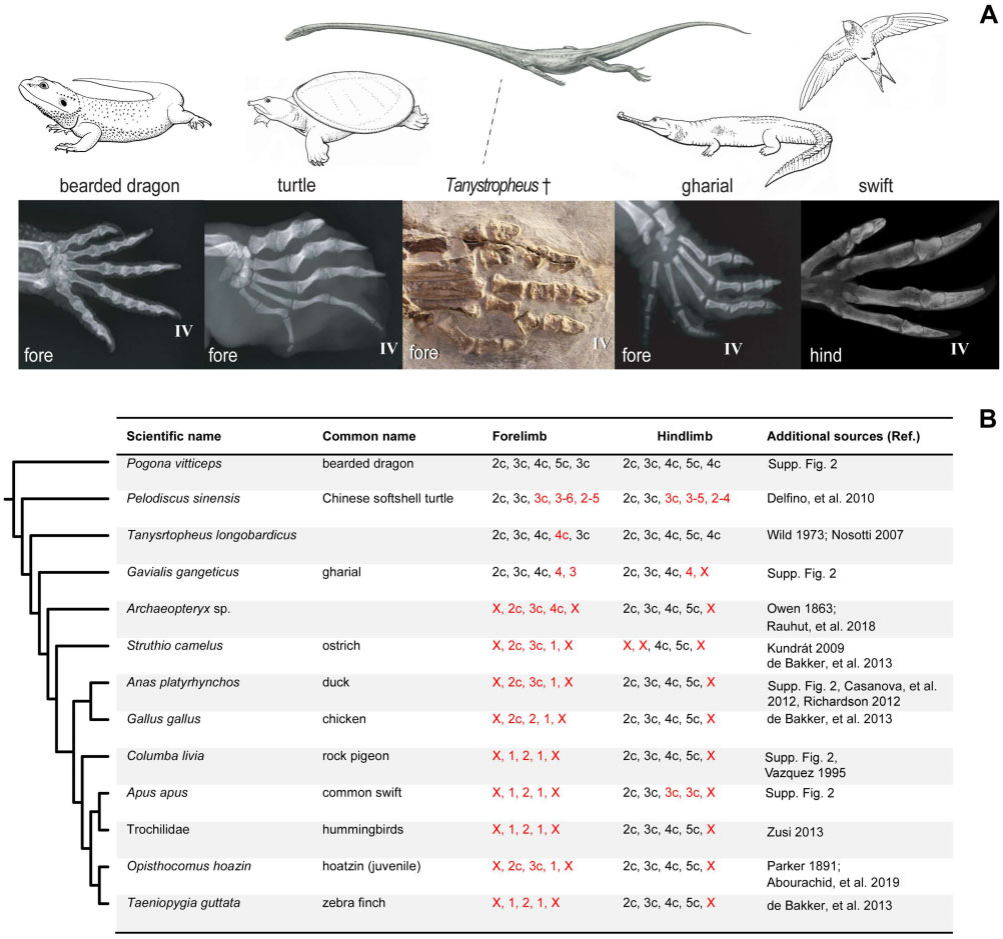
Patterns of evolutionary tinkering as expressed in adult digit phenotypes. (*A*) Skeletons (x-radiographs, except for the fossil of *Tanystropheus hydroides*); for specimen details, see supplementary table S5, Supplementary Material online; anterior to the top, distal to the right. (*B*) Details of claw and phalanx loss in selected amniote taxa. Key: red type, phalanx loss; †, extinct taxon; x, lost element; c, claw present. Phylogeny based on Chiari et al. (2012), Green et al. (2014), Jarvis et al. (2014), Stein et al. (2015), Ezcurra (2016). Drawing of *Tanystropheus* by Beat Scheffold; others illustrations by Esmée Winkel.

If, as we argue above, there is premature termination of phalanx formation in digits, can we see evidence of our proposed mechanism more widely in amniotes? To answer this question, we have surveyed phalanx number in adult amniotes. The species we consider in detail are: the Chinese soft-shelled turtle (Delfino et al. 2010), the gharial, the stem-archosaur *Tanystropheus hydroides* (Wild 1973; Spiekman et al. 2020 ), a nonarchosaurian archosauromorph, that predates the Aves-Crocodylia split within Archosauromorpha; and the common swift (fig. 8). We chose these species for two reasons. First, they all show loss of phalanges and/or claws, relative to the ancestral condition (Wagner and Gauthier 1999; Vargas et al. 2008; Xu and Mackem 2013). That condition is represented here by the bearded dragon (fig. 8). Second, they all have unambiguous digit homologies, so there is no question of a frameshift in their cases.

We summarize the loss of phalanges and claws in these and some other informative taxa in figure 8. The data show three distinct patterns: 1) loss of phalanx and claw (chicken wing digits III, IV; de Bakker et al. 2013; gharial forelimb digit IV), 2) loss of phalanx, claw persisting (common swift, hindlimb digits III, IV; *Tanystropheus hydroides* forelimb digit IV, Chinese soft-shelled turtle, fore- and hindlimb digit III), and 3) loss of the claw only (gharial, forelimb digit IV and V). In summary, there have been numerous, small changes in digit and phalanx number during amniote evolution. The significance of these findings is that they provide a further line of evidence in support of our proposal that phalanges are readily lost during evolution without any need to invoke a frameshift.

## Discussion

The frameshift hypothesis was an important milestone in the evolutionary developmental biology because it provided an ingenious means of integrating data from molecular biology, developmental biology, and paleontology. However, our *Hoxd11*, *d12*, and *d13* expression data, and our data on phalanx loss in amniotes, are not consistent with the frameshift hypothesis—or the modified frameshift hypothesis, recently proposed by Stewart et al. (2019). Both of those hypotheses assume a unique molecular signature for digit I based on posterior *Hoxd* gene and possibly *Sall1* expression. We find that the anterior boundary of *Hoxd* expression drifts during evolution across the anterior digits, making it an unstable marker. Furthermore, we find in our study that in all amniote species examined, forelimb and hindlimbs show the same expression, digit by digit. We can apply this observation to the four neognaths studied here: only hindlimb digits III and IV showed expression of *Hoxd11* and *12*; hindlimb digits I and II show no expression. Therefore, the most anterior formed digit in the wing of the chicken and other neognaths is digit II, giving a wing digit formula of II–III–IV.

One possible explanation for the discrepancy in our findings compared with those of Stewart et al. (2019) is the difference in techniques each having its pros and cons. We used in situ hybridization in 17 species which gave good spatial resolution of gene expression. In situ hybridization does not however give a quantitative readout, and only encompasses one or a few genes. By contrast, the study of Stewart et al. employed a transcriptomic approach which was quantitative, and encompassed multiple genes, but included only four species, had minimal spatial resolution and lacked a developmental series.

Our study shows that the anterior expression boundary of *Hoxd 11* and *12* extends across different digits in different species. This plasticity may due to regulatory changes. Such changes in general have been suggested to be the dominant mode of morphological evolution (Prud’homme et al. 2007; Sordino et al. 1995) suggested that changes in hox regulation may have led to the evolution of the digital plate by extending cell proliferation in the ancestral fin bud. A second example of regulatory changes in evolution is the loss of the snake hindlimb, which is due to changes in *cis* regulation *Shh* expression (Kvon et al. 2016; Leal and Cohn 2016). Alternative explanations could include changes in *trans* regulation of hox genes, for example, via evolutionary changes in the *Shh*-*Gli3* signaling pathway (reviewed by Tickle and Towers 2017).

Having argued against a frameshift, we suggest an alternative scenario: a simple loss of phalanges (and sometimes claws) taking place under natural selection. Furthermore, we have suggested a mechanism for this phalanx loss in terms of early termination of the phalanx-forming region, together with localized apoptosis. Thus, the initially strong expression of Sox9 in the phalanx forming region in wing digit IV in the chicken and duck prematurely terminate relative to the other digits. This termination of the phalanx-forming region is in contrast to its persistence in the other two wing digits, and the four digits of the hindlimb. We see the same premature termination of the phalanx forming region in the crocodile hindlimb digit V. In both cases, the premature termination of the phalanx forming region was followed by apoptosis. Interestingly, apoptosis was also one of the mechanisms proposed for digit loss in the jerboa, horse, and camel (Cooper et al. 2014). In other mammals (cows and pigs), changes in *Ptch1* expression leading to altered SHH signaling were found (Cooper et al. 2014; Lopez-Rios et al. 2014). This provides a mechanism for a change in digit phenotype without a change in digit “identity.”

Another interesting finding has come from recent phylogenetic analyses (Baron et al. 2017; Langer et al. 2017). These placed the early dinosaurs *Herrerasaurus* and *Heterodontosaurus* outside the lineage leading to birds*.* These dinosaurs both show reduction of digits IV and V in the forelimb, a fact that was used as the basis of the supposed I-II-III homology of the avian wing digits (Wagner and Gauthier 1999; Barta et al. 2018), reviewed in Towers (2018). If this new placement is correct, the trend toward reduction of digits IV and V is no longer supported and the need to postulate a frameshift may be eliminated.

This, however, leaves the forelimb phalangeal formula of *Archaeopteryx lithographica* to be explained. The three wing digits of this species have the phalangeal formula [x-2-3-4-x], see Rauhut et al. (2018). This phenotype has also been used to argue for a I-II-III homology of those digits (Wagner and Gauthier 1999). We would like to suggest an explanation which is at least as parsimonious: the digits have simply lost phalanges while retaining the claw. We show similar examples of this pattern of phalangeal loss in figure 8. Phalangeal loss without claw loss can even be observed in Aves without any question of an accompanying frame shift. For example, in the hindlimb of the swift, digits III and IV have lost one and two phalanges, respectively, while retaining the claw. Loss of the hallux (digit I) can be observed in the hindlimb of some woodpeckers and plovers, whereas some kingfishers have lost hindlimb digit III (Delacour 1951). All paleognaths studied here have lost also digit 1 in the hindlimb.

Hox genes have frequently been characterized as having coding sequences and expression patterns that are highly conserved in phylogeny, especially in the homeodomain (supplementary figs. S1 and S3, Supplementary Material online). The changes in spatial expression of the hoxd genes is therefore difficult or impossible to link to changes in their protein sequences. It seems much more likely that changes in their gene regulatory networks are responsible. This could be changes in their enhancers: its sequence, accessibility, genomic spatial organization (Fabre et al. 2015), and interactions with the proteins needed for their transcription. The deletion of half of the protein sequences and our failure to amplify *Hoxd12* from Nile crocodile cDNA (while successful with the same cDNA for *Hoxd11, Hoxd13*, and *Sox9*) could be indicative of a nonfunctioning *Hoxd12* gene in crocodilians.

One example of the conservation of expression patterns is the pattern of *Hoxd* gene expression that supposedly identifies digit I in all amniotes (Vargas et al. 2008). However, our results show considerable variation in *Hoxd* gene expression patterns in the autopod, presumably because of changes in gene regulatory networks (Woltering and Duboule 2010; Seki et al. 2017). Interestingly, we found that the expression patterns were nonetheless stable within four clades: Neognathae, Crocodylia, Iguania, and Eutherian Mammals with the exception of the mole and its morphologically specialized autopod. The digital phenotype is also stable within these clades.

A major finding of our study is the variable expression boundary of hoxd13. Previous studies have led to the view that hoxd13 is first expressed in the vicinity of the zone of polarizing activity, and then expands anteriorly to encompass all digits (Woltering and Duboule 2010). However, we find here that it remains unexpressed in the MAD in the mole hindlimb, the mouse forelimb, the turtle forelimb and hindlimb, the emu forelimb, chicken forelimb and hindlimb, and the zebra finch hindlimb (figs. 2 and 3). Furthermore, in the mole, *Hoxd11* and *Hoxd12* are expressed in digits I–V, and the *os falciforme*, whereas *Hoxd13* only extends as far anterior as digit II. This *Hoxd* expression in the *os falciforme* is surprising. There has been debate about whether this bone represents a true (autopodial) digit, a sesamoid bone or a modified carpal/tarsal element (Mitgutsch et al. 2012). The fact that it expresses *Hoxd11* and *Hoxd12*—both markers of developing digits (Nelson et al. 1996; Reno et al. 2008; Woltering and Duboule 2010)—suggest that it has the molecular signature of a true digit and not of a carpal element or sesamoid bone. It would be interesting to determine whether this might also apply to the panda’s extra “thumb” (“radial sesamoid”) (Davis 1964; Gould 1980; Endo et al. 1996).

The multiple instances of phalangeal loss that we identify in amniotes are consistent with evolutionary tinkering (Jacob 1977; Kmita and Duboule 2003; Duboule et al. 2007; Brakefield 2011). The concept of tinkering refers to evolution by means of multiple, small phenotypic changes (Jacob 1977). Among the elements of the limb skeleton, the digits are particularly subject to tinkering as Owen noted: “As the segments of each limb recede from the trunk they become subject to more extensive and varied modifications” (Owen 1835, p. 353). This might be because they are terminal characters, specified near the end of a proximal-to-distal sequence of patterning events (Richardson et al. 2004). As a result, changes in digit development may not impact downstream structures, because there are none (other than the keratinous claw). The digits are therefore less constrained to respond to selection pressures—more subject to tinkering, in other words. This study may help us understand the remarkable diversity of digit types that have evolved under many different selection pressures.

## Materials and Methods

For a list of embryos examined and number of specimens, see supplementary table S1, Supplementary Material online. All experiments conducted in the Netherlands were performed in accordance with the Experiments on Animals Act (Wet Op de Dierproeven, 2014), the applicable legislation in the Netherlands in accordance with the European guidelines (EU directive no. 2010/63/EU) regarding the protection of animals used for scientific purposes.

### Collection of Embryos

Chicken (*Gallus gallus*), duck (*Anas platyrhynchos*), greater rhea (*Rhea americana*), ostrich (*Struthio camelus*), and emu (*Dromaius novaehollandiae*) fertilized eggs were obtained from commercial breeders. Some additional emu embryos were provided by John Young (Cliff Tabin’s Lab, Department of Genetics, Harvard Medical School, USA). The zebra finch (*Taeniopygia guttata*) eggs were a gift from Carel J. ten Cate and Katharina Riebel, both of Animal Sciences and Health, Institute of Biology Leiden, Leiden University, the Netherlands. Marcel Biermans, of the same institute, provided the rock pigeon (*Columba livia*) eggs. The Nile crocodile (*Crocodylus niloticus*) eggs were a gift from La Ferme Aux Crocodiles, Pierrelatte, France and the broad-snouted caiman (*Caiman latirostris*) eggs were donated by René Hedegaard, Krokodille Zoo, Denmark.

Chinese soft-shelled turtle (*Pelodiscus sinensis*) eggs were purchased from a local farmer by Tatsuya Hirasawa and Shigeru Kuratani (RIKEN, Kobe, Japan). Hermann’s tortoise (*Testudo hermanni*) embryos were collected from a captive breeding colony by Radim Blazek. Most of the Central bearded dragon (*Pogona vitticeps*) eggs were purchased from commercial breeders, except for one clutch which was donated by Bregeta Demmer and Jordy Hol, (Purmerend, the Netherlands). The fertilized plumed basilisks (*Basiliscus plumifrons*) eggs were a gift of Oscar Maas, Reptielenhuis de Aarde, Breda, the Netherlands. The *Hemidactylus* sp. data were collected at the Museum für Naturkunde by van der Vos and Bickelmann; for more details see (van der Vos et al. 2018). The tammar wallaby (*Macropus eugenii*) embryos were from a breeding colony of wallabies caught under permit (to Marilyn Renfree) from a wild population on Kangaroo Island, South Australia. All sampling techniques and collection of wallaby tissues were approved by the University of Melbourne Animal Experimentation and Ethics Committee and conformed to the guidelines of the Australian National Health and Medical Research Council. North American least shrew (*Cryptotis parva*) were obtained of a captive breeding colony of Peter Kondrashov, the house mouse (*Mus musculus*) embryos were bred and harvested according to the Netherlands Law on Animal Testing, “Dierproefnummer, 14167u,” at Leiden University. The Iberian mole (*Talpa occidentalis*) embryos were captured in Santa Fé (Granada province, Spain) under annual permits granted to Rafael Jiménez by the Andalusian Environmental Council; animal handling was performed in accordance with the guidelines of the University of Granada’s Ethical Committee for Animal Experimentation.

After candling the eggs, we removed the embryos and transferred them to ice-cold phosphate-buffered saline (PBS) in a Petri dish. The amnion was removed, and the embryo fixed in ice-cold 4% formaldehyde in PBS at 4 °C overnight. The next day, they were dehydrated in a graded methanol series and stored in 100% methanol at −20 °C. In total, 223 embryos were hybridized in situ and analyzed (supplementary table S1, Supplementary Material online).

### Probes

The mouse *Hoxd13* and *Hoxd11* probes were a gift from Denis Duboule. All other probes were made in-house and their sequences deposited at GenBank (www.ncbi.nlm.nih.gov/genbank/, last accessed May 25, 2021). See supplementary table S2, Supplementary Material online for the list of probes used. All in situ hybridization of bird embryos was done with chicken *Hoxd13, Hoxd12*, and *Hoxd11* probes. The caiman was hybridized with a Nile crocodile probe. We made many unsuccessful attempts to amplify crocodilian *Hoxd12* (including designing nine forward and ten reserve primers). For the plumed basilisk, we used bearded dragon probes. Concerning *Hoxd13*: the mouse probe was used for all mammals examined, the chick probe for birds, and the crocodile probe for the crocodile and turtle. All other experiments were done with species-specific probes. The *Sox9* probes are identical to the ones used in de Bakker et al. (2013).

For the probes synthesized in-house, we isolated total RNA from an embryo using Trizol (Invitrogen) and carried out reverse transcription using SuperScript III (Invitrogen). PCR was performed on these templates using specific primers, and the PCR products were cloned in the TOPOTA-PCRII vector (Invitrogen). The inserted amplicons were checked by a PCR with M13-pUC primers located on the TOPOTA-PCRII plasmid and checked on an agarose gel. When they were of the right size they were sent for Sanger sequencing. After confirming the sequence results by BLAST searching, the positive results were used as templates for making the digoxigenin-labeled antisense RNA probes.

### Whole Mount In Situ Hybridization

The embryos were rehydrated through a graded methanol series, lightly digested with proteinase K (20–40 µg/ml in PBS) for 20 min and postfixed in 4% formaldehyde in PBS after several washes in PBST (PBS pH 7.2 with 0.1% Tween-20). This was followed by a prehybridization step at 60 °C for at least 3 h or until the embryo had sunk. The hybridization mixture consisted of: 50% formamide, 2% Boehringer blocking powder, 5× SSC (from 20× standard sodium citrate buffer, 3 M sodium chloride, 0.3 M sodium citrate, pH 7), 1 mg/ml total RNA, 50 μg/ml heparin, 0.1% Triton X-100, 0.1% CHAPS (3-[(3-cholamidopropyl) dimethylammonio]-1-propanesulfonate) and 5 mM EDTA (ethylenediaminetetraacetic acid). After the prehybridization mix was removed, we added 400 ng/ml specific probe to fresh hybridization mixture preheated to 60 °C. The embryos were incubated in this mix at 60 °C overnight with slow shaking. The next day the specific probe mixture was removed, collected and stored at −20 °C for reuse.

Several stringent washes were done at 60 °C to remove nonspecifically bound probe [2× SSC, 0.1% CHAPS, 50% formamide]; [2× SSC 0.1% CHAPS]; [0.2× SSC, 0.1% CHAPS]. After washing several times at room temperature with TBST (0.1 M tris [tris (hydroxymethyl)aminomethane] buffered saline, pH 7.5, 0.1% Tween-20) the embryos were preincubated with heat inactivated 10% sheep serum in TBST for 90 min at room temperature followed by overnight incubation with sheep antidigoxigenin conjugated to alkaline phosphatase (Roche; 1:5,000 dilution in 10% sheep serum in TBST at 4 °C overnight). Next day, the nonspecifically bound antibodies were washed away by several washes with TBST of which the last one was overnight at 4 °C. The embryos were brought to a higher pH by washing them in NTT buffer (0.1 M sodium chloride, 0.1 M Tris/HCl, 0.1% Tween-20, pH 9.5). The enzyme reaction of alkaline phosphate with BM purple (Roche) as substrate results in a blue precipitate. The development of the stain was checked regularly and stopped by washing several times in TBST, removing the substrate and chromogens, and lowering the pH.

### Wholemount TUNEL Staining

The embryos were collected and fixed as for in situ hybridization rehydrated through a graded methanol series, washed in TBST (0.1 M tris-buffered saline containing 0.1% Tween), pretreated with proteinase K (40 µg/ml, 20 min at room temperature), washed in TBST, postfixed with 4% formaldehyde in PBST, washed in TBST followed by a wash in the TdT buffer (30 mM Tris/HCl, 140 mM Na cacodylate, 0.1% Triton pH 7.2), preincubated in the reaction mix (70 nM digoxigenin-labeled dUTP, 400 nM ATP, and 1 U/ml terminal transferase in the enzyme buffer) at 4 °C without the cofactor CoCl_2_ which was added to the reaction mix (1 mM end-concentration) after 1 h. With CoCl_2_ added, the embryos were incubated at room temperature overnight. The reaction was stopped by adding 200 mM EDTA (ethylenediaminetetraacetic acid), pH 7.0, 1/10 of the reaction volume. The digoxygenin-labeled nucleotides were localized with a standard antidigoxigenin antibody conjugated to alkaline phosphatase procedure followed by staining with BM-purple.

### TUNEL Staining of Sections

Wings of embryos fixated and dehydrated as above were, rinsed in 100% ethanol followed by Histo-Clear (National Diagnostics, Atlanta, GA), embedded in paraffin wax, sectioned at 7 μm and mounted on silane-coated slides (VWR International B.V, Amsterdam). The dewaxed slides were treated with 20 μg/ml proteinase K in PBS for 20 min, postfixed in 4% formaldehyde in PBS. The TUNEL reaction took place at 37 °C for 2 h in a similar reaction mix as above but with a higher concentrations of digoxygenin-labeled dUTP (3.5 μM) and ATP (20 μM) and CoCl_2_ already added to the mix. The reaction was stopped by rinsing the slides in 20 mM EDTA in PBS. TUNEL-positive cells were localized with a standard antidigoxigenin antibody conjugated to alkaline phosphatase followed by staining with BM-purple. As a counter stain we used 0.1% neutral red.

### Measurement of the Volume of Digit IV in the Chicken Embryo

Two chicken embryos of stage 35 and two of stage 36 were fixed in 5% trichloroacetic acid (TCA) in water and stained with Alcian blue for cartilage. The stained fore- and hindlimbs were photographed with a microruler for calibration when taking measurements using FIJI image analysis software. For each specimen, the width and length of all four digits IV, namely all phalanges but not including the metacarpal or metatarsal, were measured five times. The measurements were taken from the level of the metacarpophalangeal joint to the tip of digit IV. The width of the forelimb phalanx and the second phalanx in the hindlimb were measured (as shown in fig. 7*H* and *I*) ). From these measurements, volume estimations of the digits were calculated with the following formula: ((W × W)/4) × L × π (W= width, L = length) in µm^3^. The averages of these five measurements with their standard deviations are shown as a bar graph in figure 7.

### Radiography

Digital radiographs were made with a Hewlett-Packard, type Faxitron M110/Faxitron Cabinet X-ray System 43855C. For the origins and accession numbers of the specimens radiographed, see supplementary table S3, Supplementary Material online.

### Protein Alignment

The mouse is considered to be one of the best annotated genomes. We used mouse genomic sequences of *Hoxd11*, *Hoxd12*, and *Hoxd13* to query Uniprot with Blast searches (McGinnis and Madden 2004; Johnson et al. 2008) to find the Hoxd orthologs in a selection of species. The hits were also checked, with their annotation, in the databases of Uniprot and NCBI to confirm their identity. The quality of some genomes is low and their computational gene annotations have low confidence. For this reason, low quality and partial sequences were excluded. For the same reason we also replaced some of our species with closely related species, namely: *Testudo hermanni* with *Gopherus agassizii*; *Basiliscus plumifrons* with *Anolis carolinensis*; and *Crocodylus niloticus* with *Alligator mississippiensis*, *A. sinensis* and *Crocodylus porosus*. For the alignments of the three Hoxd genes we used AliView (Larsson 2014). The phylogeny is based on (Chiari et al. 2012; Green et al. 2014; Jarvis et al. 2014; Stein et al. 2015).

### Data Availability

The data underlying this article will be shared on reasonable request to the corresponding author.

## Supplementary Material

Supplementary data are available at *Molecular Biology and Evolution* online.

## Supplementary Material

msab150_Supplementary_Data
