# Supplementary material for: Selection on Phalanx Development in the Evolution of the Bird Wing
Source: Mol Biol Evol. 2021 Jun 23;38(10):4222–37. doi: 10.1093/molbev/msab150 (PMC8476175; doi:10.1093/molbev/msab150)
Supplement: msab150_Supplementary_Data [file msab150_supplementary_data.pdf]

Supplementary Information

Supplementary Figure 1 **Protein alignment of *Hoxd11* of selected amniotes.** Both the N- and C-terminals of *Hoxd11* are conserved, but the C-terminal containing the DNA binding domain (homeobox) is 100% conserved. The intervening sequence is less conserved and includes significant insertions in *Pogona vitticeps, Mus musculus, Talpa occidentalis* and *Macropus eugenii. P. vitticeps* has also a significant deletion and *Struthio camelus* has some bad sequencing. The sequence differences between species are probably not only due to evolutionary changes but might also be due to sequencing errors, software prediction mistakes and missing data in the sequences retrieved from the databases.

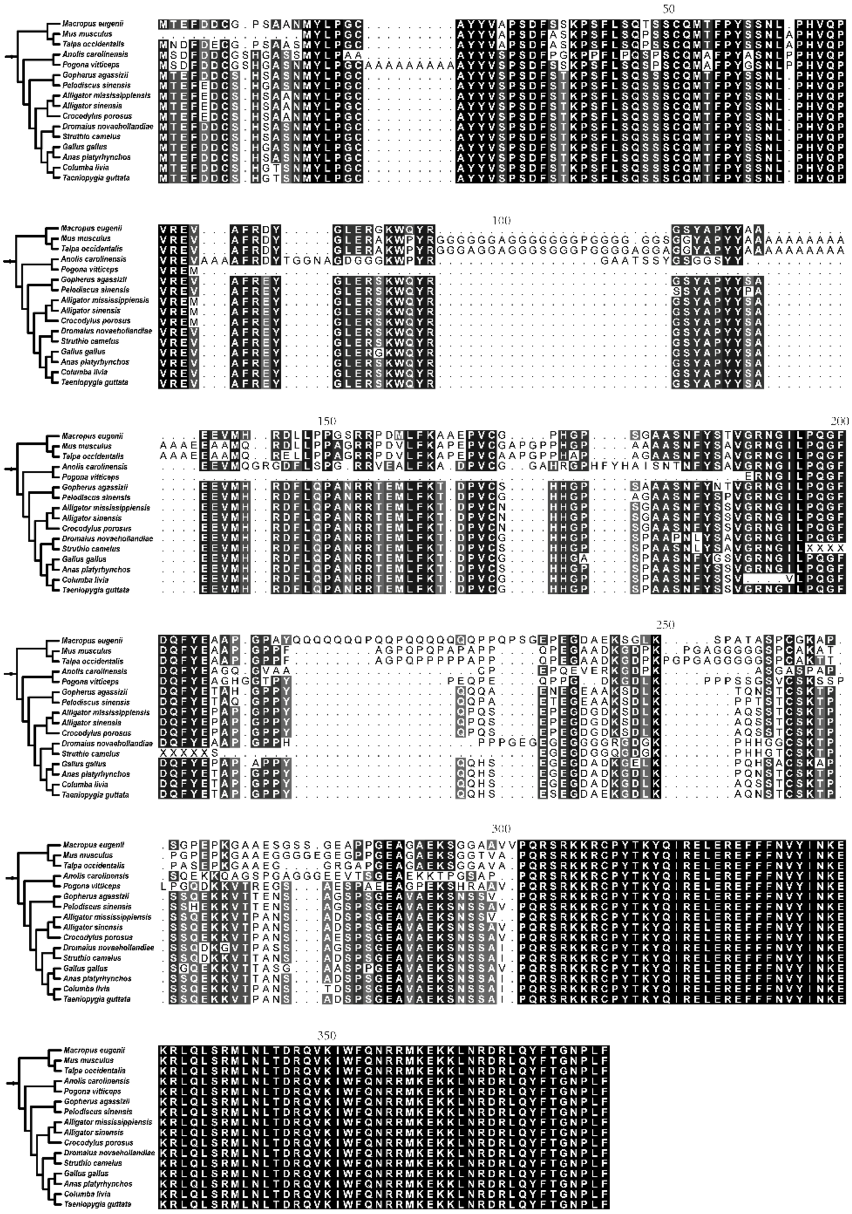

Supplementary Figure 2 **Protein alignment of *Hoxd12* of selected amniotes**. Both the N- and C-terminals are more conserved than the intervening region. The three crocodilians (*Alligator mississippiensis, A. sinensis* and *Crocodylus porosus*) have a deletion of more than 150 amino acids - about half of the normal length of the protein. The sequence differences between species are probably not only due to evolutionary changes but might also be due to sequencing errors, software prediction mistakes and missing data in the sequences retrieved from the databases.

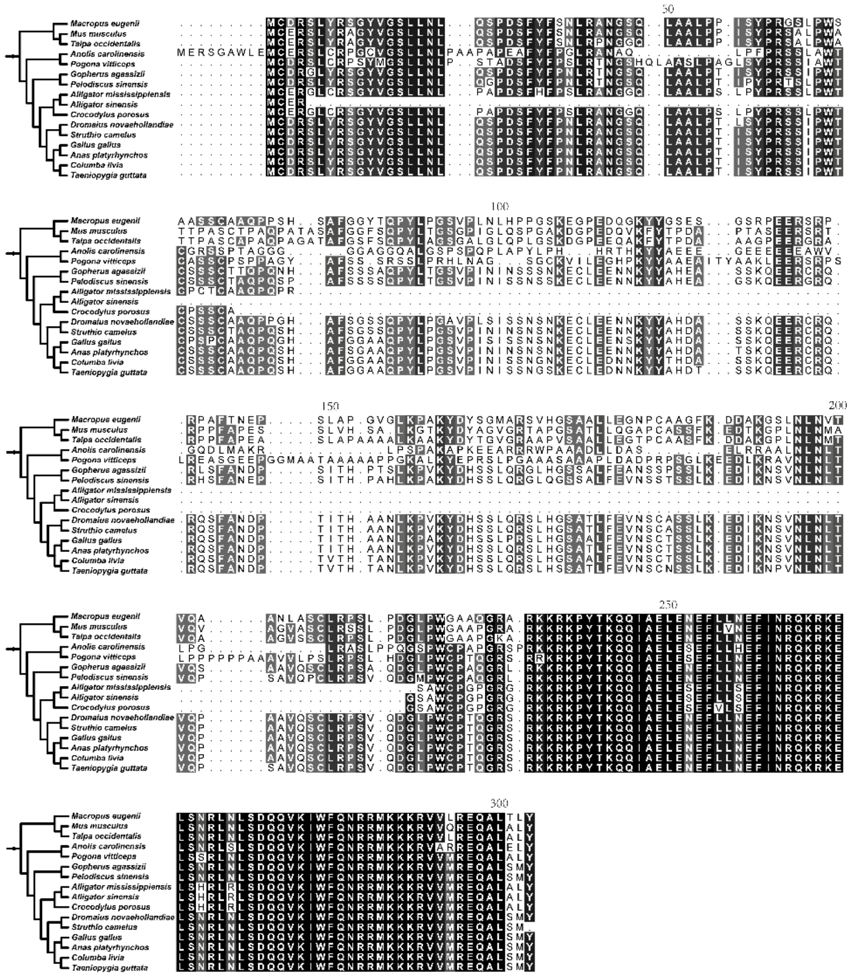

Supplementary Figure 3 **Protein alignment of Hoxd13 of selected amniotes.** The C-terminal is highly conserved except for short poly-A tracts in the two lizards (*Anolis carolinensis* and *Pogona vitticeps*). The amino terminal is more divergent, partly due to truncation of the sequence in several species, in particular *Pogona vitticeps*, the three crocodilians, and three birds *Struthio camelus*, *Anas platyrhynchos* and *Columba livia*. The sequence differences between species are probably not only due to evolutionary changes but might also be due to sequencing errors, software prediction mistakes and missing data in the sequences retrieved from the databases.

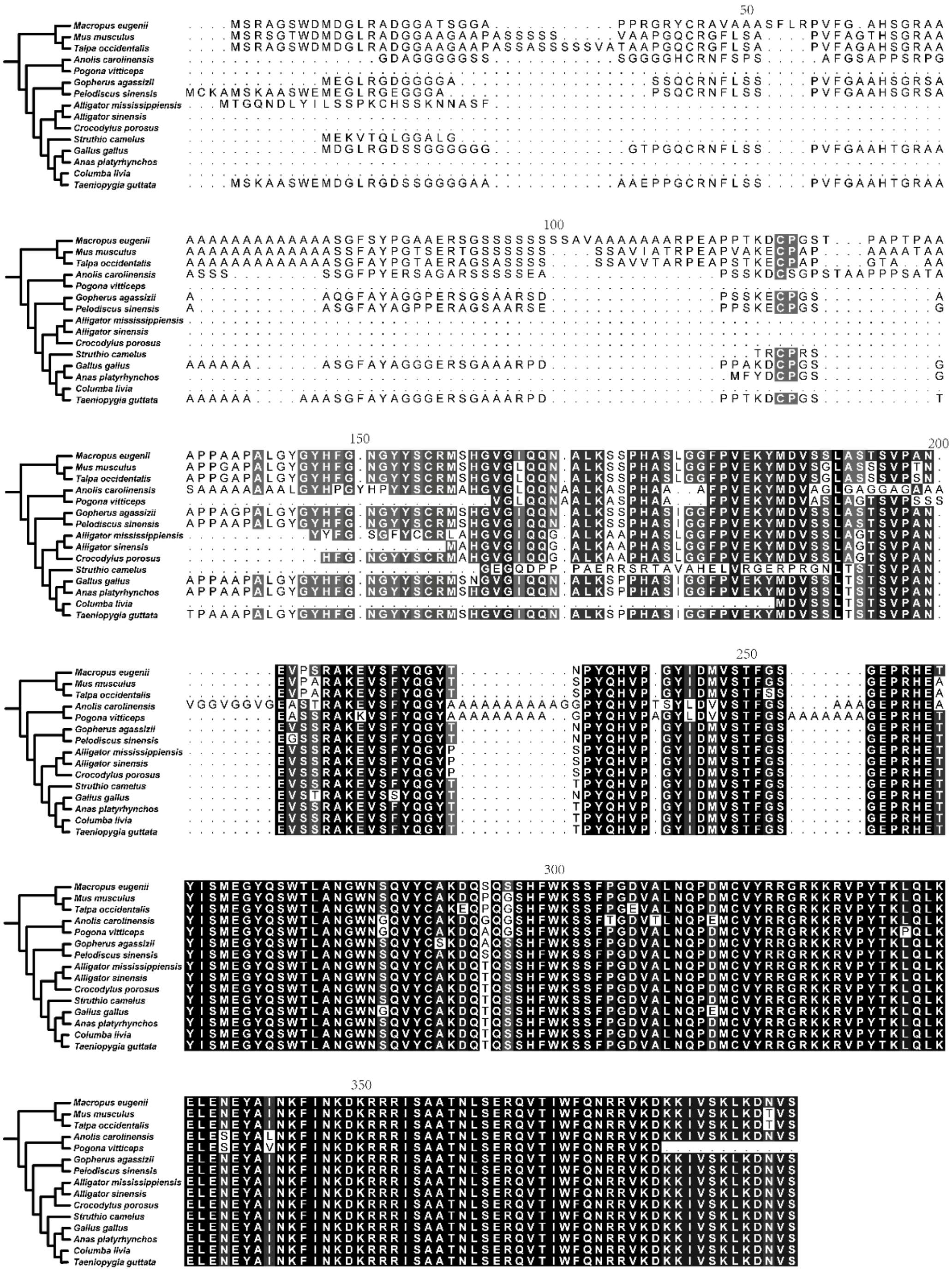

Supplementary Figure 4 **Skeletal morphology of adult limbs**. a-f, radiographs; g-h, skeletal preparations. Text under figures gives the phalangeal formula; x, digit lost; c, claw present; red numerals and *, digit changed compared to ancestral state.

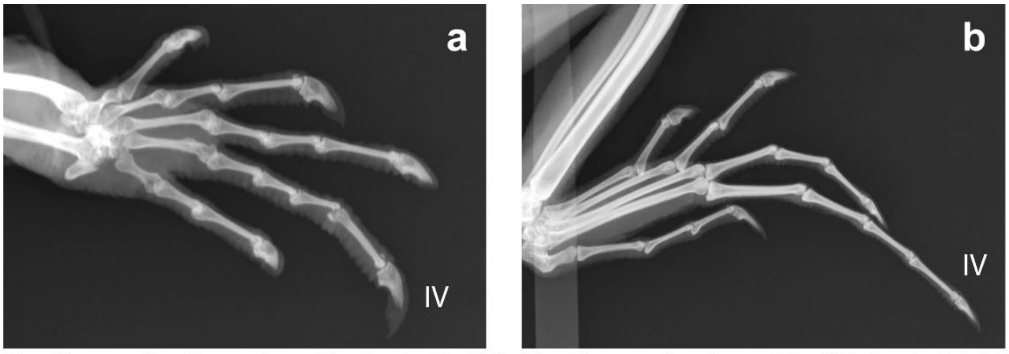

Basiliscus plumifrons fore [2c-3c-4c-5c-3c] *Basiliscus plumifrons* hind [2c-3c-4c-5c-4c]

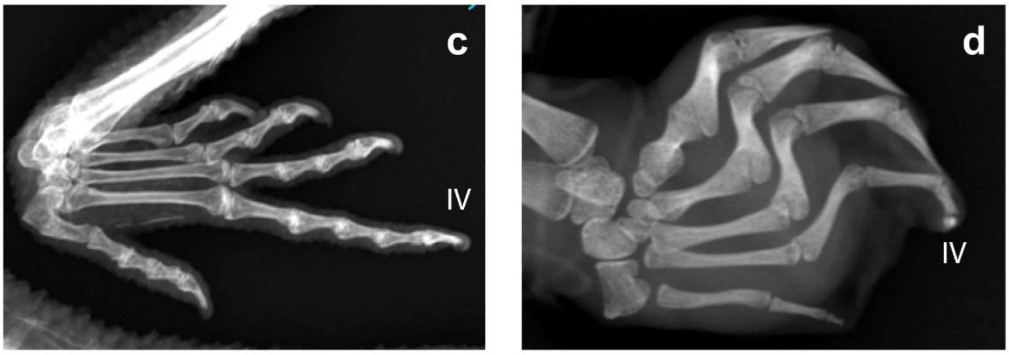

Pogona vitticeps hind [2c-3c-4c-5c-4c] *Pelodiscus sinensis* hind [2c-3c-3c-4-2]

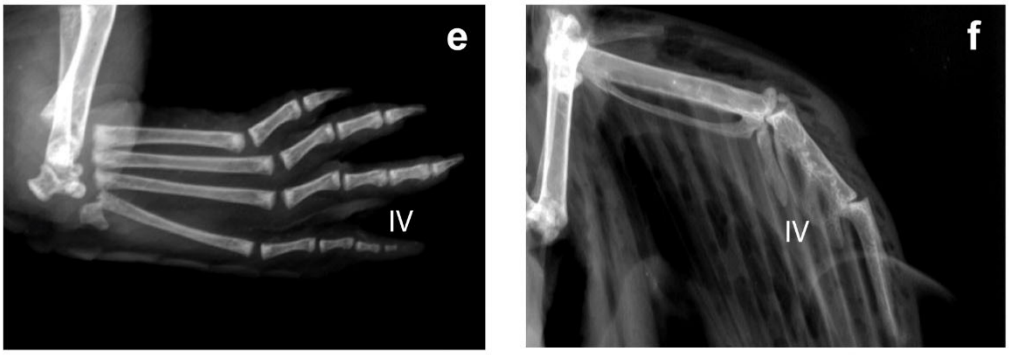

Gavialis gangeticus hind [2c-3c-4c-4-x] *Apus apus* fore [x-1-2-1-x]

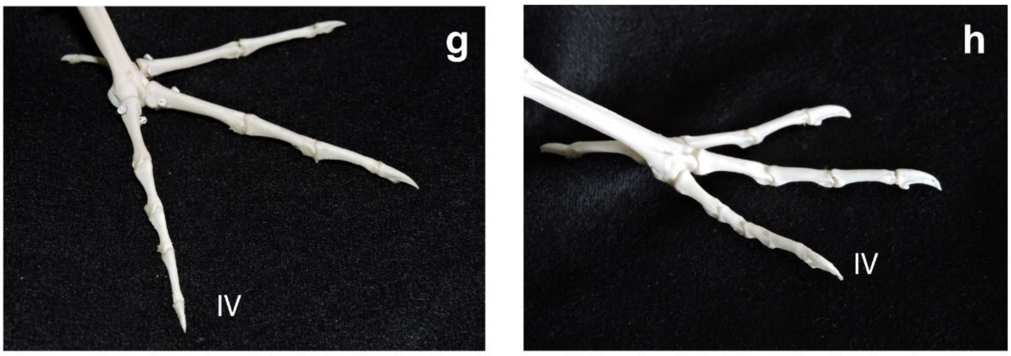

Anas platyrhynchos hind [2c-3c-4c-5c-x] *Columba livia* hind [2c-3c-4c-5c-x]

Supplementary Figure 5 **Whole embryo *in situ* hybridisation of *Hoxd11* and *Hoxd12* in the wallaby and mole**. This figure confirms that these probes are specific. Line drawings by Esmée Winkel.

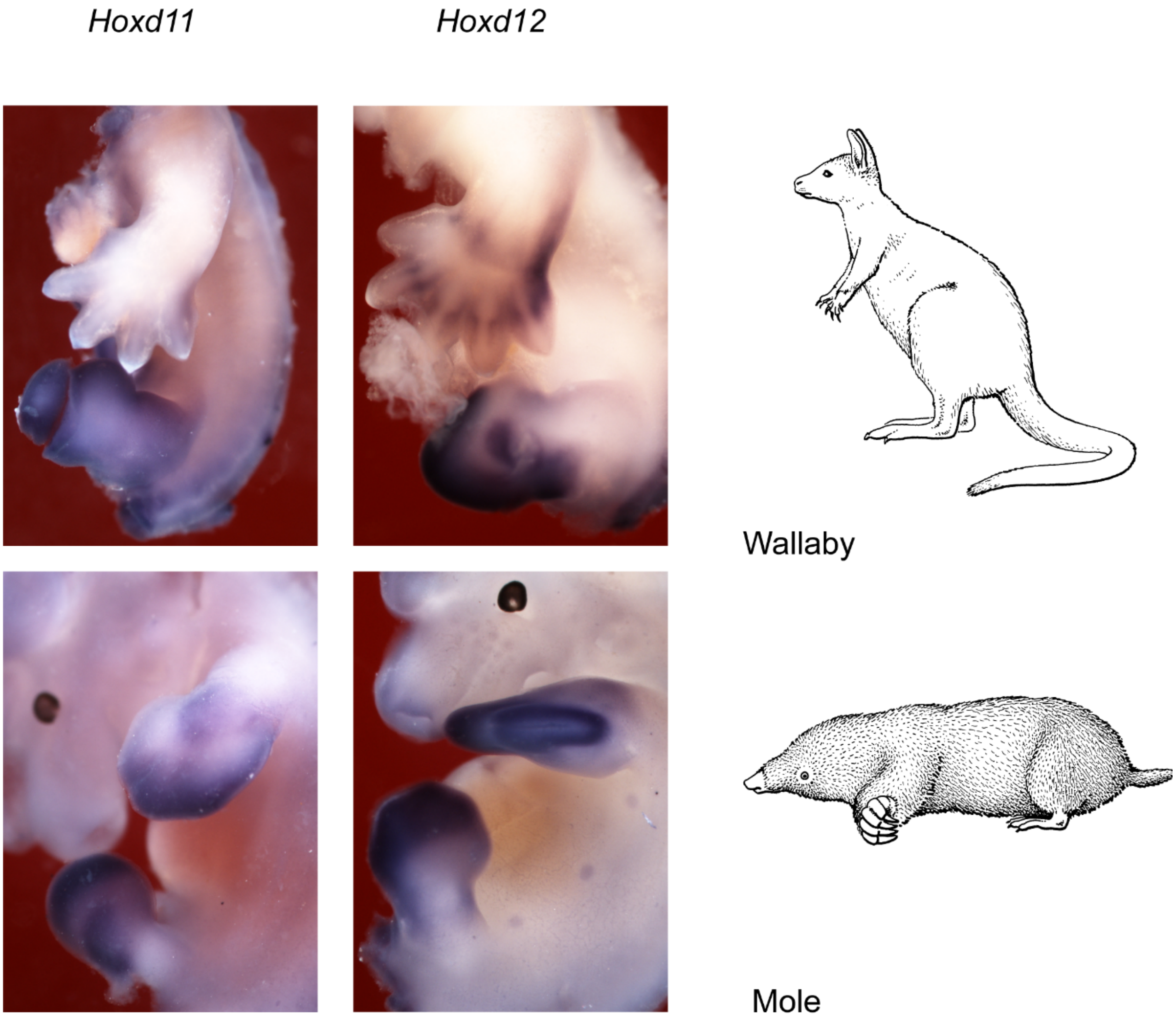

Supplementary Table 1 **Number of experiments and embryos used**. One experiment is a staining of a pair of fore- and hind limbs. For a more accurate comparison we used contralateral limbs of the same individual to compare different gene expression patterns for *Hoxd11* and *Hoxd12*, so the total of embryos is less than the total of expression studies.

Scientific name	*Hoxd13*	*d12*	*d11*	*Sox9*	TUNEL	Alcian blue	experiments	embryos
Mus musculus	7	7	8				22	19
Cryptotis parva		2	2				4	2
Talpa occidentalis	5	10	12				27	19
Macropus eugenii		5	4				9	6
Hemidactylus sp.	1						1	1
Basiliscus plumifrons		1	1				2	1
Pogona vitticeps		5	7				12	7
Pelodiscus sinensis	2	4	3				9	7
Testudo hermanni		5	4				9	5
Crocodylus niloticus	2		11	3	2		18	18
Caiman latirostris		1	1				2	1
Struthio camelus	2	3	13				18	16
Dromaius novaehollandiae	2	4	10				16	14
Rhea americana		5	5				10	7
Gallus gallus	14	7	6	4	21	4	56	54
Anas platyrhynchos	2	4	6	4	3		19	16
Columba livia		2	2				4	2
Taeniopygia guttata	16	6	5				27	28
**Total**	**52**	**68**	**100**	**14**	**26**	**4**	**265**	**223**

Supplementary Table 2 **Probes used with their GeneBank (NCBI) accession numbers**. Note: (*): even after many attempts, including designing 9 forward and 10 reserve primers, we did not succeed in amplifying *Hoxd12* of the Nile crocodile. *Hoxd11* homeobox d11, *Hoxd12* homeobox d12, *Hoxd13* homeobox d13 and *Sox9* SRY (sex determining region Y)-box 9.

species	gene	forward primers	reverse primer	length (bp)	accession number
Mus musculus	*Hoxd11*	Gift from Denis Duboule lab			NM_008273
Talpa occidentalis	*Hoxd11*	ccctacaccaagtaccagatcc	ccattttactagaaaccatttattcac	689 (CDS 204 + 485 5' UTR)	KX896995
Macropus eugenii	*Hoxd11*	atgaccgagtttgacgactg	tgaaatattggagtcgatctctg	895 (CDS)	KX896994
Pogona vitticeps	*Hoxd11*	atgagtgactttgacgattgcg	tccctgttcagtttcttttcttcatcc	575 (CDS)	KX896993
Pelodiscus sinensis	*Hoxd11*	ccgggtgcgcttattacgtc	cggtcggtgaggtttagcat	700 (CDS)	KX896992
Testudo hermanni	*Hoxd11*	atgactgagtttgacgattgca	aaacaaggggtttccagtgaa	837 (CDS)	MN486487
Crocodylus niloticus	*Hoxd11*	agcctcttgtgcaatagatggatc	catccttcgattctggaaccag	859 (5'UTR 76 + 783 CDS)	JF317537
Gallus gallus	*Hoxd11*	gttcaacgcattgaagcctcc	tgccggtcagtgaggttgag	803 (5'UTR 51 + 752 CDS)	JF317555
Mus musculus	*Hoxd12*	atgtgtgagcgcagtctctacagagc	ggtcgctgaggttcagcctgtt	727 (CDS)	KX896999
Talpa occidentalis	*Hoxd12*	atgtgtgagcgcagtctctacagagc	gctggtcgctgaggttcagcct	833 (CDS)	KX896998
Macropus eugenii	*Hoxd12*	atgtgcgatcgcagtctct	ggaaccagattttgacctgtt	741 (CDS)	KX897000
Pogona vitticeps	*Hoxd12*	gacccagttacatgggctctct	ttcgtcggttctggaacca	876 (CDS)	KX896997
Pelodiscus sinensis	*Hoxd12*	acttgcggactaatggcagt	atagatagcgcctgctcacg	712 (CDS)	KX896996
Testudo hermanni	*Hoxd12*	atgtgtgaccgcggtctcta	ctagtacatagaaagcgcctgct	801 (CDS)	MN486488
Gallus gallus	*Hoxd12*	tgttggaaatgtgtgatcgcag	cgcctgctcgcgcattac	794 (5'UTR 8 + 786 CDS)	JF317555
Mus musculus	*Hoxd13*	Gift from Denis Duboule lab			NM_008275
Hemidactylus sp.	*Hoxd13*	agccgcgccacgaaacctaca	tccttcagcttggagacg at	370 (CDS)	MW082608
Pogona vitticeps	*Hoxd13*	ggtcgggctacagcagaacgc	tgtccttgacgcggcggtt	612 (CDS)	MW187869
Crocodylus niloticus	*Hoxd13*	gctacgggtatcactttggcaa	aacgttgtctttcagtttggagacta	631 (CDS)	JF317539
Gallus gallus	*Hoxd13*	gctatgggtatcactttggcaa	gagactattttcttatccttcaccct	650 (CDS)	JF317556
Crocodylus niloticus	*Sox9*	atgaatctcctggaccccttca	catctgcacaacgccgagct	837 (CDS)	JQ717196
Dromaius novaehollandiae	*Sox9*	atgaatctcctggaccccttca	cgaactcgttgacgtcgaaggt	886 (CDS)	JQ717195

Supplementary Table 3 Source data and references for skeletal phenotypes illustrated in Figure 1.

scientific name	digit formula fore	digit formula hind	reference
Macropus eugenii	2c, 3c, 3c, 3c, 3c	X, 3c, 3c, 3c, 3c	(Chew, et al. 2012)
Mus musculus	2c, 3c, 3c, 3c, 3c	2c, 3c, 3c, 3c, 3c	(Woltering and Duboule 2010; Parmenter, et al. 2016)
Cryptotis parva	2c, 3c, 3c, 3c, 3c	2c, 3c, 3c, 3c, 3c	(Mitgutsch, et al. 2012)
Talpa occidentalis	F, 2c, 3c, 3c, 3c, 3c	F, 2c, 3c, 3c, 3c, 3c	(Mitgutsch, et al. 2012)
Pogona vitticeps	2c, 3c, 4c, 5c, 3c	2c, 3c, 4c, 5c, 4c	
Basiliscus plumifrons	2c, 3c, 4c, 5c, 3c	2c, 3c, 4c, 5c, 4c	
Pelodiscus sinensis	2c, 3c, 3c, 3-6, 2-5	2c, 3c, 3c, 3-5, 2-4	(Delfino, et al. 2010)
Testudo hermanni	1c, 2c, 2c, 2c, 1c	1c, 2c, 2c, 2c, 1	(Crumly and Sanchez-Villagra 2004; Hitschfeld, et al. 2008)
Caiman latirostris	2c, 3c, 4c, 4, 3	2c, 3c, 4c, 4, X	(Reynolds 1897; Iungman, et al. 2008) fig. 48 p. 264
Crocodylus niloticus	2c, 3c, 4c, 4, 3	2c, 3c, 4c, 4, X	(de Bakker, et al. 2013)
Dromaius novaehollandiae	X, X, 3c, X, X	X, 3c, 4c, 5c, X	(Maxwell and Larsson 2007, 2009; de Bakker, et al. 2013)
Rhea Americana	X, 2c, 2, 1, X	X, 3c, 4c, 5c, X	(Maxwell and Larsson 2009)
Struthio camelus	X, 2c, 3c, 1, X	X, 3c, 4c, X, X	(Reynolds 1897; Kundrát 2009; de Bakker, et al. 2013)
Anas platyrhynchos	X, 2c, 3c, 1, X	2c, 3c, 4c, 5c, X	
Galllus gallus	X, 2c, 2, 1, X	2c, 3c, 4c, 5c, X	
Columba livia	X, 1, 2, 1, X	2c, 3c, 4c, 5c, X	
Taeniopygia guttata	X, 1, 2, 1, X	2c, 3c, 4c, 5c, X	(de Bakker, et al. 2013)

Supplementary Table 4 **Volumes (μm^3 x 10^6) of Alcian blue-stained cartilage** Measurements are from fore- and hindlimb digits IV in two developing chicken stages 35 (GgHJ46 and GgHJ78) and 36 (GgMB26 and GgMB25) with standard deviation and the ratio of fore- and hindlimb volumes.

	volumes digit IV (μm^3 x 10^6)	standard deviation (μm^3 x 10^6)	ratio hind/fore
GgHJ46 left forelimb	4.34	0.05	15.49
GgHJ46 left hindlimb	67.29	5.20	
GgHJ46 right forelimb	4.05	0.59	14.72
GgHJ46 right hindlimb	59.66	3.95	
GgHJ78 left forelimb	6.01	0.71	20.90
GgHJ78 left hindlimb	125.73	6.82	
GgHJ78 right forelimb	4.40	1.10	15.93
GgHJ78 right hindlimb	70.26	5.98	
GgMB26 left forelimb	7.63	1.03	21.21
GgMB26 left hindlimb	162.04	3.91	
GgMB26 right forelimb	6.11	0.31	26.19
GgMB26 right hindlimb	160.23	8.98	
GgMB25 left forelimb	5.74	0.49	29.73
GgMB25 left hindlimb	170.74	17.08	
GgMB25 right forelimb	3.83	0.43	54.39
GgMB25 right hindlimb	208.81	20.15	

Supplementary Table 5 **Details of adult limbs examined**. The specimen numbers relate to the collection of the respective institutes. n/a (not available) unnumbered specimen.

scientific name	common name	institute	collection	specimen number
Pogona vitticeps	bearded dragon	Institute Biology Leiden, Leiden University, the Netherlands	alcohol	n/a
Basiliscus plumifrons	plumed basilisk	Naturalis Biodiversity Center, Leiden, the Netherlands	alcohol	RMNH.RENA.5957
Pelodiscus sinensis	Chinese softshell turtle	Naturalis Biodiversity Center, Leiden, the Netherlands	alcohol	RMNH.RENA.40855
Tanystropheus longobardicus		Paläontologisches Institut und Museum, Universität Zürich	fossil	PIMUZ T 2817
Gavialis gangeticus	gharial	Naturalis Biodiversity Center, Leiden, the Netherlands	alcohol	RMNH.RENA.3198
Anas platyrhynchos	duck (mallard)	Institute Biology Leiden, Leiden University, the Netherlands	skeleton	705
Apus apus	common swift	Naturalis Biodiversity Center, Leiden, the Netherlands	frozen	n/a
Columba livia	rock pigeon	Institute Biology Leiden, Leiden University, the Netherlands	skeleton	915

Supplementary References

Chew, Yu, Pask, Shaw, Renfree. 2012. HOXA13 and HOXD13 expression during development of the syndactylous digits in the marsupial Macropus eugenii. Bmc Developmental Biology 12.

Chiari, Cahais, Galtier, Delsuc. 2012a. Phylogenomic analyses support the position of turtles as the sister group of birds and crocodiles (Archosauria). BMC.Biol. 10:65.

Chiari, Cahais, Galtier, Delsuc. 2012b. Phylogenomic analyses support the position of turtles as the sister group of birds and crocodiles (Archosauria). Bmc Biology 10.

Crumly, Sanchez-Villagra. 2004. Patterns of variation in the phalangeal formulae of land tortoises (Testudinidae): Developmental constraint, size, and phylogenetic history. Journal of Experimental Zoology Part B-Molecular and Developmental Evolution 302b:134-146.

de Bakker, Fowler, den Oude, Dondorp, Navas, Horbanczuk, Sire, Szczerbinska, Richardson. 2013. Digit loss in archosaur evolution and the interplay between selection and constraints. Nature 500:445-448.

Delfino, Fritz, Sanchez-Villagra. 2010. Evolutionary and developmental aspects of phalangeal formula variation in pig-nose and soft-shelled turtles (Carettochelyidae and Trionychidae). Organisms Diversity & Evolution 10:69-79.

Ezcurra. 2016. The phylogenetic relationships of basal archosauromorphs, with an emphasis on the systematics of proterosuchian archosauriforms. Peerj 4.

Green, Braun, Armstrong, Earl, Nguyen, Hickey, Vandewege, St John, Capella-Gutierrez, Castoe, et al. 2014. Three crocodilian genomes reveal ancestral patterns of evolution among archosaurs. Science 346:1335-+.

Hamburger, Hamilton. 1951. A series of normal stages in the development of the chick embryo. J.Morphol. 88:49-92.

Hiscock, Tschopp, Tabin. 2017. On the formation of digits and joints during limb development. Dev Cell 41:459-465.

Hitschfeld, Auer, Fritz. 2008. Phalangeal formulae and ontogenetic variation of carpal morphology in Testudo horsfieldii and T, hermanni. Amphibia-Reptilia 29:93-99.

Huang, Trofka, Furusawa, Norrie, Rabinowitz, Vokes, Mark Taketo, Zakany, Mackem. 2016. An interdigit signalling centre instructs coordinate phalanx-joint formation governed by 5'Hoxd-Gli3 antagonism. Nat Commun 7:12903.

Iungman, Pina, Siroski. 2008. Embryological development of Caiman latirostris (Crocodylia : Alligatoridae). Genesis 46:401-417.

Jarvis, Mirarab, Aberer, Li, Houde, Li, Ho, Faircloth, Nabholz, Howard, et al. 2014. Whole-genome analyses resolve early branches in the tree of life of modern birds. Science 346:1320-1331.

Johnson, Zaretskaya, Raytselis, Merezhuk, McGinnis, Madden. 2008. NCBI BLAST: a better web interface. Nucleic Acids Res 36:W5-W9.

Kundrát. 2009. Primary chondrification foci in the wing basipodium of *Struthio camelus* with comments on interpretation of autopodial elements in Crocodilia and Aves. J Exp.Zool B Mol.Dev Evol. 312:30-41.

Larsson. 2014. AliView: a fast and lightweight alignment viewer and editor for large datasets. Bioinformatics 30:3276-3278.

Maxwell, Larsson. 2009. Comparative ossification sequence and skeletal development of the postcranium of palaeognathous birds (Aves: Palaeognathae). Zoological Journal of the Linnean Society 157:169-196.

Maxwell, Larsson. 2007. Osteology and myology of the wing of the Emu (Dromaius novaehollandiae), and its bearing on the evolution of vestigial structures. Journal of Morphology 268:423-441.

Mitgutsch, Richardson, Jimenez, Martin, Kondrashov, de Bakker, Sanchez-Villagra. 2012. Circumventing the polydactyly 'constraint': the mole's 'thumb'. Biology Letters 8:74-77.

Parmenter, Gray, Hogan, Ford, Broman, Vinyard, Payseur. 2016. Genetics of Skeletal Evolution in Unusually Large Mice from Gough Island. Genetics 204:1559-+.

Reynolds. 1897. The Vertebrate Skeleton. Cambridge: University Press
Stein, Brown, Mooers. 2015. A molecular genetic time scale demonstrates Cretaceous origins and multiple diversification rate shifts within the order Galliformes (Aves). Molecular Phylogenetics and Evolution 92:155-164.
Suzuki, Hasso, Fallon. 2008. Unique SMAD1/5/8 activity at the phalanx-forming region determines digit identity. Proc.Natl.Acad.Sci.U.S.A 105:4185-4190.
van der Vos, Stein, Di-Poï, Bickelmann. 2018. Ontogeny of Hemidactylus (Gekkota, Squamata) with emphasis on the limbs. Zoosystematics and Evolution 94:195-209.
Woltering, Duboule. 2010. The Origin of Digits: Expression Patterns versus Regulatory Mechanisms. Developmental Cell 18:526-532.
